# Playing position and match location affect the number of high-intensity efforts more than the quality of the opposition in elite football players

**DOI:** 10.5114/biolsport.2024.133669

**Published:** 2023-12-20

**Authors:** Ryland Morgans, Daeeun Kweon, Ben Ryan, Wonwoo Ju, Piotr Zmijewski, Rafael Oliveira, Sigrid Olthof

**Affiliations:** 1 School of Sport and Health Sciences, Cardiff Metropolitan University, Cardiff, UK; 2 Brentford FC Football Research Centre, Brentford FC, London, UK; 3 High Performance Group, Korea Football Association, Republic of Korea; 4 Jozef Pilsudski University of Physical Education in Warsaw, 00-809 Warsaw, Poland; 5 Research and Development Center Legia Lab, Legia Warszawa, Poland; 6 Research Centre in Sports Sciences, Health and Human Development, 5001–801 Vila Real, Portugal; 7 Sports Science School of Rio Maior – Instituto Politecnico de Santarem, 2040–413 Rio Maior, Portugal; 8 Life Quality Research Centre, 2040–413 Rio Maior, Portugal; 9 Research Institute for Sport and Exercise Sciences, Liverpool John Moores University, Liverpool, UK

**Keywords:** Accelerometry-based variables, Position-specific demands, High-intensity actions, Soccer, Developing players

## Abstract

This study aimed to examine the impact of playing position (PP), match location (ML), and opposition standard (OS) on team and individual acceleration (ACC) and deceleration (DEC) efforts. Fifty professional football players were monitored across 24 English Premier Development League matches during the 2020/21 season. High-intensity ACC and DEC thresholds were set at > +3 m · s^−2^ and < -3 m · s^−2^, respectively. Players were divided into five PPs: centre backs (CB; n = 68), full-backs (FB; n = 24), centre midfielders (CM; n = 54), wide midfielders (WM; n = 15), centre forwards (CF; n = 27). Opposition standard was categorised as Top (1^st^–4^th^), Middle (5^th^–9^th^), and Bottom (9^th^–13^th^) based on final league ranking of the study season. Each match location was classified as Home or Away. One way analysis of variance (ANOVA) and a multivariate ANOVA analysed the independent effect of PP, ML and OS on ACC and DEC efforts, and the interaction of all contextual factors, respectively. Acceleration efforts were affected by PP and ML. FB performed 22% more ACC than WM. All players performed 6% more ACC actions during home matches compared to away fixtures. DEC efforts were only affected by PP, with FB and CM executing 26% and 32% greater DEC efforts than CB, respectively. When playing against top or middle teams at home, CB, CM, and CF tended to perform more high-intensity actions than when playing away. In contrast, when playing against top teams at home, FB and WM performed fewer high-intensity actions than when playing away. Playing position and ML affected ACC and DEC actions but not OS.

## INTRODUCTION

Acceleration (ACC) and deceleration (DEC) actions in football elicit mechanical demands, contributing 7–10% and 5–7% to the total workloads of the elite players during match-play, respectively, regardless of playing position (PP) [1]. Moreover, the number of ACC actions during competitive match-play is 3–8 times greater than that of sprint actions [2], while DEC actions occur as frequently as ACC actions inducing an even higher mechanical load [1]. Nonetheless, existing studies have predominantly focused on measuring the total distance covered and high-intensity running (physiological load) in isolation whilst not considering ACC and DEC actions (biomechanical load) [1, 3]. This potentially leads to underestimating players’ biomechanical load since most ACC actions have been reported to not reach high-intensity running thresholds [2, 4].

Such underestimation of players’ biomechanical load in matches may increase the potential risk of injury, given that ACC and DEC actions are highly associated with neuromuscular fatigue [5]. Specifically, high rates of force development and highly coordinated neural activation are required to execute ACC and DEC activities, leading to induced muscle damage, mechanical fatigue and reducing neural drive [6]. Furthermore, ACC actions are known to be metabolically more demanding than running at a constant velocity [4]. Additionally, high eccentric braking actions that occur when decelerating rapidly seem to constitute the greatest volume of mechanical load per metre in comparison to other high-intensity activities [1]. Thus, quantifying ACC and DEC efforts during match-play to minimise and identifying the injury risk has recently been examined [6].

Accurately and objectively quantifying players’ match actions is required to improve the understanding of players’ workloads during match-play. With the emergence of modern motion-tracking technologies, the biomechanical loads and corresponding physiological responses of high-intensity actions can be quantified in an objective manner [7]. Such an advance in technology has provided sports scientists and/or team’s performance staff with practical insight into designing individualised recovery plans and position-specific training programmes by monitoring players’ workloads [8]. However, previous studies that examined ACC and DEC efforts have reported contradictory findings [1, 8–13]. This discrepancy may be attributed to the variation in high-intensity actions according to various contextual factors [13–19], which highlights the high match-to-match variability of high-intensity actions in football [20]. Thus, an investigation of ACC and DEC efforts should incorporate contextual factors to provide a comprehensive understanding of those match-play actions.

The physical demands of football matches differ according to PP [21]. Recently, it was reported that wide midfielders covered the greatest distance during high-intensity running, with central defenders covering the least distance during match-play [22]. Moreover, Ingebrigtsen, Dalen [23] found that sprinting distance by wide midfielders was ~290 m, which is more than twice as much than covered by central defenders per match. Nonetheless, studies that examined the effect of PP on the number of ACC and DEC actions are scarce in the existing literature, while conflicting findings were observed between studies [2, 23, 24]. This necessitates more investigations to determine whether positional differences exist in ACC and DEC actions in elite development football.

The match location (ML) has been regarded as an important contextual factor that affects physical demands [3, 25, 26]. However, it seems to affect the running demands not as much, but can impact ACC and DEC. To illustrate, external load variables showed a tendency to be higher during home matches although no significant differences were found [25]. Another study revealed that ML did not influence the external load but confirmed that PP had a major impact [3]. This was also confirmed in a study that found high-intensity actions during match-play are less affected by ML compared to quality of opposition and match outcome [26]. Furthermore, less high-intensity ACC and DEC actions were performed in home matches versus away matches [22]. With the contrasting findings between running and acceleration demands and limited information on acceleration demands, it seems unclear if ML affects the number of ACC and DEC actions during match-play.

The opposition standard (OS) has been considered as another contextual factor that may influence physical demands during matchplay, but the direction of influence seems inconclusive. When playing against higher-standard teams, elite football players covered a greater distance by high-intensity running (HIR), which consists of high-speed running (HSR) (≥ 19.8–25.1 km/h^−1^) and sprinting (> 25.2 km/h^−1^) [4], whereas other research highlighted that increased running demands were observed when playing against similar-level opponents [27]. A recent study observed that DEC (< –4 m∙s^−2^) showed higher values against top-level compared with middle- and bottom-level opponents [28]. As a limited number of studies have investigated the effect of OS on high-intensity ACC and DEC actions [12, 26, 28, 29], it restricts any definitive conclusions assessing the effect of those actions.

In the current literature, information is relatively limited on the effect of PP, ML, and OS on high-intensity ACC and DEC, and no study has investigated its effect on those actions in the English Premier Development (U23) League. Understanding the acceleration and deceleration demands in match-play supports the identification and development of talented players. Therefore, the current study aimed to determine the effects of ML, OS, and PP on the number of high-intensity ACC and DEC actions performed. A secondary aim was to examine any team and positional biomechanical demand differences. It was hypothesised that; a) positional differences appear in ACC and DEC actions, b) less high-intensity ACC and DEC actions would be performed playing at home compared to playing away, and c) greater high-intensity ACC and DEC efforts are produced when competing against higher OS compared to lower OS.

## MATERIALS AND METHODS

### Participants & Data Sample

This observational study involved professional football players participating in the English Premier Development League. A total of 50 elite football players (age 27 ± 5 years; height 181 ± 6 cm; body mass 75 ± 8 kg) from one academy (U23) team were monitored in the 2020/2021 season. The team played 24 matches in the English Premier League Development League. After excluding participants who played less than 90 minutes (n = 110) from the analysis, 189 player observations were included for further data analysis. Goalkeepers were excluded from the investigation due to the specific nature of the match activity and low running demands [23]. The professional club from which the participants volunteered approved the secondary data analyses. All data collected resulted from normal analytical procedures regarding player monitoring over the competitive season. Gatekeeper consent was obtained for secondary data analysis. Data from all players were compiled into a repository, and the Research Ethics Committee of Liverpool John Moores University approved secondary data analyses (Reference number: U22SPS2076).

### Contextual Factors

Players were assigned to one of five playing positions as match physical demands for these differ significantly [24]. Match observations were classified according to playing positions as follows: centre backs (CB; n = 68), full-backs (FB; n = 24), centre midfielders (CM; n = 54), wide midfielders (WM; n = 15) and centre forwards (CF; n = 27). The study team consistently played in a 1-4-4-2 tactical system. To establish OS, all teams in the English Premier League Development League during the 2020/2021 season were categorised as Top (1^st^–4^th^, player observations n = 64), Middle (5^th^–9^th^, n = 67), and Bottom (9^th^–13^th^, n = 57), with the reference team being classified as Middle based on the final league table of the study season [30]. Regarding ML, each match was categorised according to the location in which the game occurred (home, n = 95 and away, n = 93). To examine the independent effect of contextual factors, OS, ML, and PP were analysed separately. The factors OS, ML, and PP were also combined to determine the interactive effect.

### Data Collection Procedure

The biomechanical loads were consistently monitored across the study seasons during all matches using player tracking technology with an 18 Hz global positioning system (GPS) system (Apex Pod, version 4.03, 50 g, 88 × 33 mm; STATSports; Northern Ireland, UK). StatSports devices had good reliability across sessions for high accelerations (CV = 0.0 to 4.0%), good to moderate for low accelerations (CV = 2.3 to 7.8%), moderate accelerations (CV = 1.7 to 7.5%), low decelerations (CV = 1.6 to 8.0%) and high decelerations (CV = 1.1 to 6.6%), and good to poor for moderate decelerations (CV = 4.8 to 10.2%) [31]. All devices were activated 30-minutes before data collection to allow the acquisition of satellite signals and to synchronise the GPS clock with the satellite’s atomic clock [32]. To avoid potential inter-unit error, each player wore the same device during the study period [33], although the present GPS system has previously reported excellent inter-unit reliability [34]. Specifically designed vests were used to hold the devices, located on the player’s upper torso, and anatomically adjusted to each player. The GPS signal quality and horizontal dilution of position (HDOP) was connected to a mean number of 21 ± 3 satellites, range 18–23, while HDOP for all seasons ranged between 0.9–1.3.

On completion of each match, GPS data were extracted using proprietary software (Apex, 10 Hz version 4.3.8, STATSports Software; Northern Ireland, UK) as software-derived data is a more simple and efficient way for practitioners to obtain data in an applied environment, with no differences reported between processing methods (software-derived to raw processed) [35]. Biomechanical loads during all matches were obtained from the GPS not from the accelerometer. Furthermore, the internal processing of the GPS units utilised the Doppler shift method to calculate both distance and velocity data which is shown to display a higher level of precision and less error compared with data calculated via positional differentiation [36]. STATSports provided written permission to allow all data to be used for research purposes. Variables analysed were selected based on previous publication [37] and in practical settings are commonly utilised by analysts in elite football. The following physical variables were quantified: the number of high-intensity accelerations (> 3 m ∙s^−2^ with minimum duration of 0.5 s); the number of high-intensity decelerations (< -3 m ∙ s^−2^ with minimum duration of 0.5 s) [31].

### Statistical Analysis

Descriptive statistics were utilised in summarising all demographic findings of this study. Data are reported as the mean ± standard deviation (SD). Statistical analyses were conducted using IBM SPSS Statistics for Mac OS X, version 27 (IBM Corp., Armonk, N.Y, USA). Data normality was checked using a Shapiro-Wilk test. One way analysis of variance (ANOVA) was selected to analyse the independent effect of OS, ML, and PP on ACC and DEC efforts. Bonferroni posthoc tests were then applied when a significant difference appeared to identify any localised effects. A multivariate ANOVA (MANOVA) was utilised to determine the interaction effect of all contextual factors. Effect size (ES) for the meaningfulness of the difference was determined based on the following criterion: (≤ 0.2 = trivial), (> 0.2–0.6 = small), (> 0.6–1.2 = moderate), (> 1.2–2.0 = large), and (> 2.0–4.0 = very large) [38]. Statistical significance was set at P ≤ 0.05.

## RESULTS

**FIG. 1:**
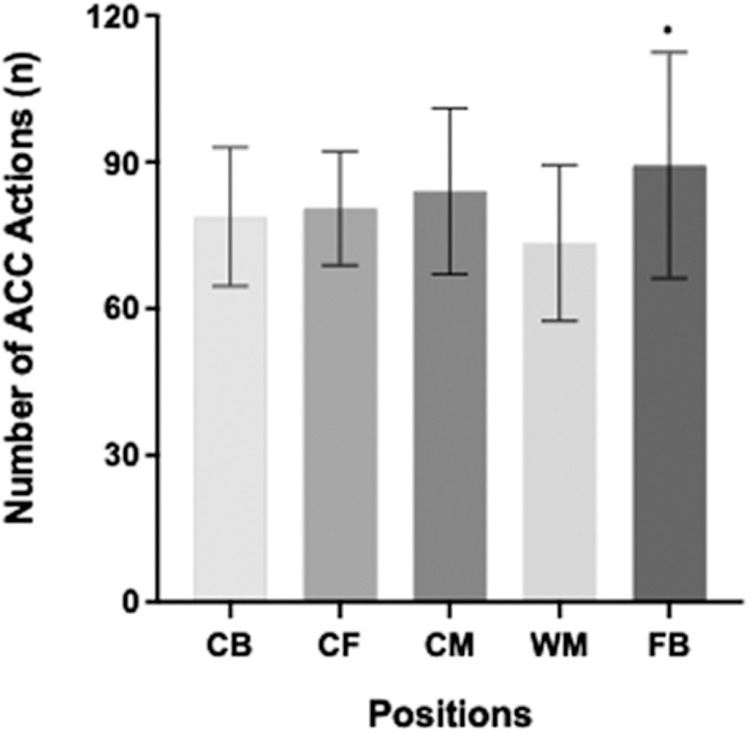
The mean number of acceleration (ACC) actions performed by different playing positions. CB: centre backs; CF: centre forwards; CM: central midfielders; WM: wide midfielders; FB: full-backs. *Greater number of actions performed than WM (P < 0.05).

The average number of ACC actions was significantly affected by different PP (P < 0.05; Figure 1). The highest mean ACC efforts were observed for FB (n = 89), followed by CM, CF, CB, and WM (n = 84, 81, 79, and 73, respectively). Post-hoc analysis revealed that FB performed 22% greater ACC actions than WM (P < 0.05; ES: 0.8), but no differences were observed for other positions.

**FIG. 2:**
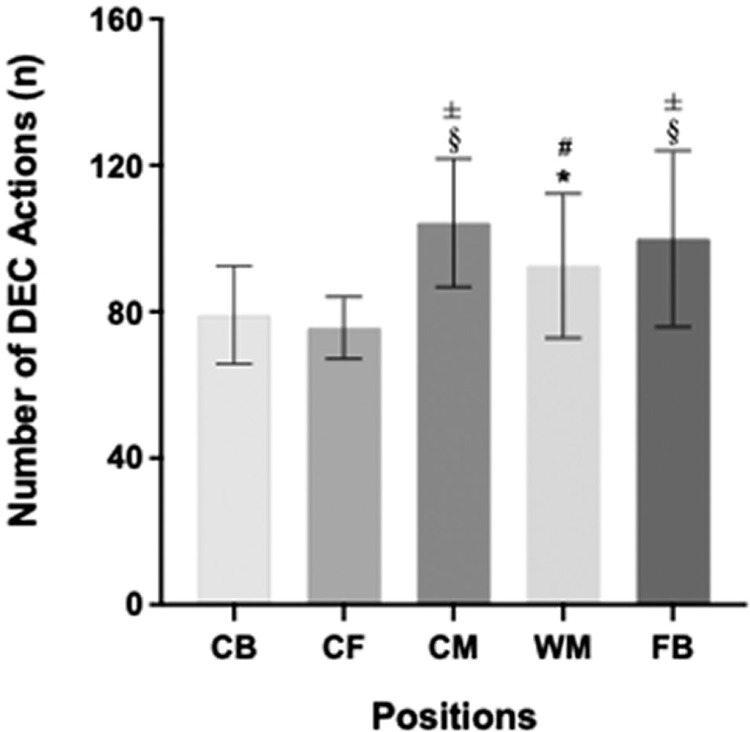
The mean number of deceleration (DEC) actions performed by different playing positions. CB: centre backs; CF: centre forwards; CM: central midfielders; WM: wide midfielders; FB: full-backs. ‡ Greater number of actions than CB (P < 0.001); § Greater number of actions than CF (P < 0.001); # Greater number of actions than CB (P < 0.05); * Greater number of actions performed than CF (P < 0.05).

Further, the mean number of DEC efforts were significantly affected by PP (P < 0.001; Figure 2). The highest DEC effort was observed in CM (n = 104), followed by FB, WB, CB, and CF (n = 100, 92, 79, and 76, respectively). Post-hoc analysis revealed that the FB, CM, and WM produced more DEC actions than the CB and CF. Specifically, the FB and CM performed 26% (P < 0.001; ES: 1.2) and 32% (P < 0.001; ES: 1.6) more DEC efforts than the CB, respectively. Similarly, FB and CM produced 32% (P < 0.001; ES: 1.4) and 38% (P < 0.001; ES: 1.9) more DEC actions than the CF, respectively. Wide midfielders executed 17% and 22% more DEC efforts compared to CB and CF, respectively (P < 0.05; ES: 0.9–1.3).

**FIG. 3:**
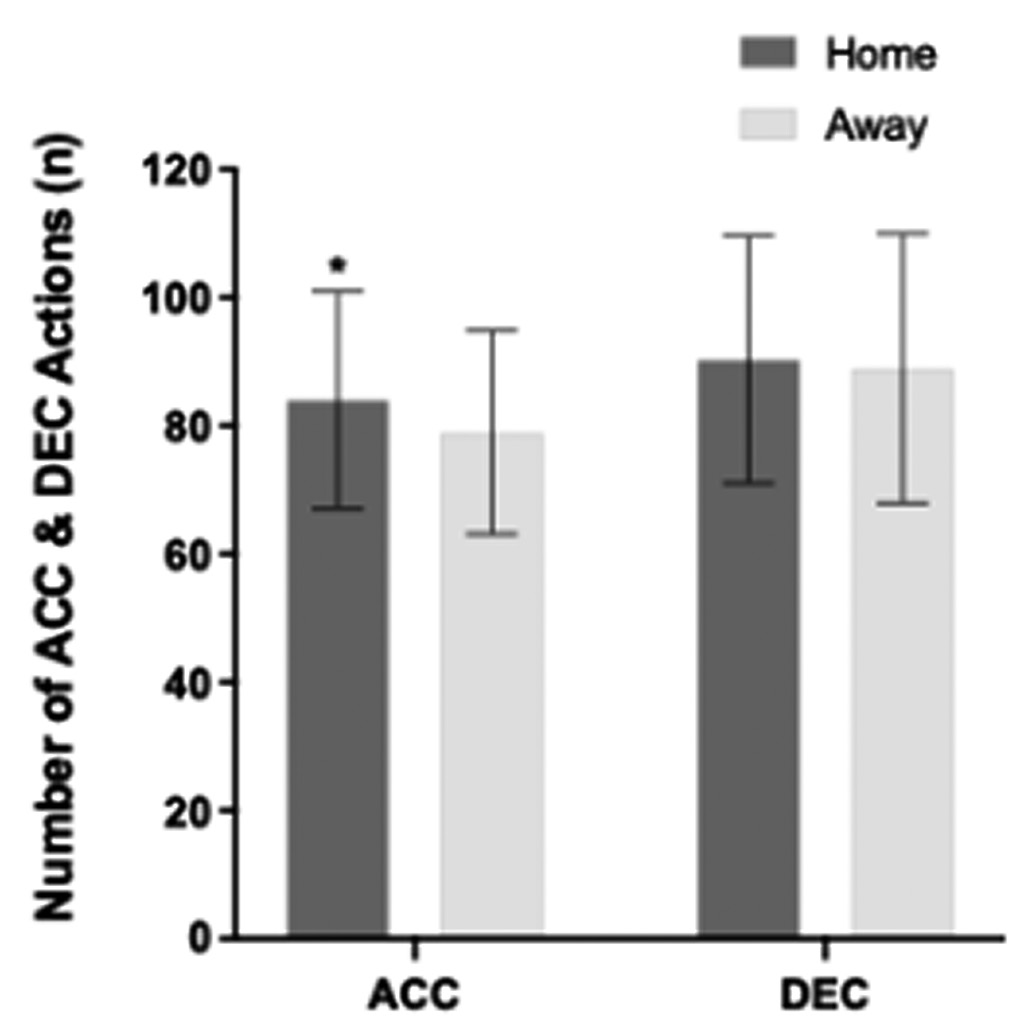
The mean number of acceleration (ACC) and deceleration (DEC) actions performed by all playing positions when considering match location. *Greater number of actions performed in home than away matches (P < 0.05).

The mean number of ACC efforts were significantly affected by ML (P < 0.05; Figure 3). All players performed 6% more ACC actions in home matches compared to away matches (P < 0.05; ES: 0.3); whereas a non-significant difference (2%) was found in the DEC actions between home and away matches (P = 0.635; ES: 0.1).

**FIG. 4:**
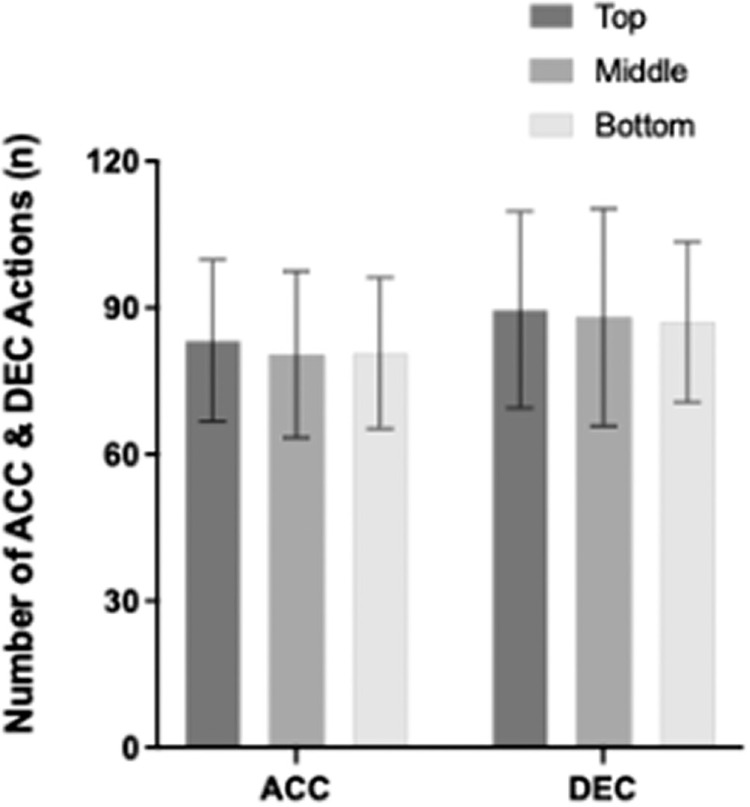
The mean number of acceleration (ACC) and deceleration (DEC) actions from all playing positions whilst considering opponent standards.

The mean number of ACC (P = 0.569) and DEC (P = 0.166) efforts were not affected by OS (Figure 4). The largest number of ACC and DEC actions were performed by the study team in matches against top teams compared to those against middle and bottom teams, but this fails to reach significance.

Table 1 illustrates the mean number of ACC and DEC efforts considering OS and ML across various PP. Against top teams, CB and CF performed more ACC actions at home than away, but FB performed less ACC and DEC actions. Against middle teams, CM performed more ACC and DEC actions at home than away. When playing against bottom teams at home, WM performed 33% more ACC actions (P < 0.05; ES: 2.0) than against bottom teams at away. However, WM performed less DEC actions against top teams at home than away.

**TABLE 1:** The descriptive statistics of the average acceleration and deceleration efforts when considering opposition standard and match location across different playing positions.

Contextual Factors			Acceleration			Deceleration		

Paying positions	Opposition standard	Match locations	Mean efforts (N)	Standard deviation (± SD)	Sample size (N)	Mean efforts (N)	Standard deviation (± SD)	Sample size (N)
**CB**	Top	Home	86.7*	15.8	13	84.1	13.4	13
		Away	72.8	12.8	11	79.7	14.1	11

	Middle	Home	76.6	15.9	12	78.1	16.4	12
		Away	77.5	11.0	12	73.8	11.1	12

	Bottom	Home	79.2	15.3	11	82.4	13.9	11
		Away	79.2	12.2	9	76.0	10.5	9

**CF**	Top	Home	86.5*	1.0	4	77.8	4.1	4
		Away	72.0	10.7	5	69.6	12.9	5

	Middle	Home	86.0	12.2	5	76.0	6.9	5
		Away	73.8	11.1	5	78.0	9.3	5

	Bottom	Home	86.3	12.2	4	76.8	8.3	4
		Away	81.3	13.4	4	76.5	7.5	4

**CM**	Top	Home	88.5	14.9	11	107.4	11.7	11
		Away	81.1	20.3	9	112.8	14.9	9

	Middle	Home	92.9*	13.2	9	119.2**	15.4	9
		Away	75.4	15.8	10	89.5	20.2	10

	Bottom	Home	78.8	18.6	8	91.5*	10.4	8
		Away	88.1	16.3	7	105.3	8.8	7

**WM**	Top	Home	88.5	3.5	2	97.0**	14.1	2
		Away	72.0	0	1	117.0	0	1

	Middle	Home	70.0	25.6	3	94.3	40.3	3
		Away	66.0	9.9	2	87.5	19.1	2

	Bottom	Home	85.0*	13.5	3	88.7	4.7	3
		Away	64.0	11.7	4	88.5	16.4	4

**FB**	Top	Home	79.0**	26.5	3	90.3**	6.8	3
		Away	101.6	22.2	5	115.0	27.2	5

	Middle	Home	88.4	36.9	5	91.8	25.6	5
		Away	91.8	5.1	4	106.0	17.8	4

	Bottom	Home	91.5	13.4	3	92.5	36.1	3
		Away	81.6	21.5	5	97.0	28.9	5

**Total**	Top	Home	86.7	14.6	33	92.4	15.9	33
		Away	79.7	18.9	31	94.6	24.8	31

	Middle	Home	83.4	20.3	34	92.1	25.4	34
		Away	77.3	13.0	33	83.9	18.0	33

	Bottom	Home	81.6	15.2	29	85.6	13.7	29
		Away	81.0	15.9	29	88.5	18.9	29

	Total	Home	84.0*	17.0	95	90.3	19.4	95
		Away	79.0	15.9	93	88.9	21.0	93

Abbreviations = CB: central back; CF: centre forward; CM: centre midfielder; WM: wide midfielder; FB: full-back. The symbol

‘*’ denotes significant difference compared to away matches (P < 0.05);

‘**’ denotes significant differences compared to away matches (P < 0.001).

## DISCUSSION

This study aimed to determine the independent effect of PP, ML, and OS on the number of high-intensity ACC and DEC actions performed by a team competing in the English Premier Development League. Further, all contextual variables (i.e., PP, ML, and OS) were also combined to determine the interaction effect on the number of high-intensity ACC and DEC actions. The main findings of the current study were: (1) positional variations in the number of ACC and DEC actions were evident; (2) the number of ACC actions were 6% greater during home matches compared to away matches; and (3) ACC and DEC efforts were not affected by OS.

### Number of Acceleration and Deceleration

In the current study, the average total number of high-intensity ACC and DEC actions performed during match-play were 82 and 90, respectively. These findings support Vigh-Larsen, Dalgas [8], who also highlighted similar numbers of ACC and DEC actions, 81 and 84 respectively. However, other research have reported more ACC and DEC actions than the current study [1, 11]. For instance, Australian A-league players accelerated and decelerated more frequently, with on average 113 and 148 times during match-play [11]. Contrary to this, Dalen, Jørgen [1] found relatively small numbers of ACC and DEC actions were performed (76 and 54, respectively) in elite Norwegian players. This may partly be attributed to the cultural differences (i.e., preferred formations or playing styles) between different European football leagues [19]. Additionally, this may be due to different speed thresholds (i.e., 2.78 vs. 2.0 vs. 3.0 m ∙ s^−2^) for high-intensity ACC and DEC actions adopted by previous studies. Thus, caution should be applied when making comparisons between studies. While differences in arbitrary thresholds between studies seem negligible, researchers should utilise common arbitrary thresholds for high-intensity ACC and DEC actions [6], since this would allow researchers and practitioners to directly and easily compare match-day ACC and DEC profiles between studies and various cohorts.

### Playing Position

This study has highlighted that high-intensity ACC actions were affected by the PP, which aligns with previous studies [1, 26]. It has been frequently reported that CB perform the lowest number of ACC activities during match-play, whilst WM execute the greatest compared to all other positions [23, 24]. However, Varley and Aughey [2] found that the greatest ACC effort was observed in FB, agreeing with a finding from the present study. That said, the present study also reported a contrasting result. Wide midfielders performed the lowest number of ACC actions, which was significantly lower than those performed by FB. Thus, it might be speculated that the tactical principle of the reference team during match-play pre-disposed FB to perform the lowest number of ACC. For instance, WM players perform in a more central pitch area than a lateral area when in possession. This tactical movement of WM attracts the opposition FB inside, creating more space on the flanks for FB to perform HIR (e.g., overlapping runs), which may be attributed to more high-intensity ACC performed by FB in this study. In contrast, when out of possession, FB may need to quickly recover from the opposition half to hold a dominant defensive position. Such a tactical evolution and various playing systems in modern football may require FB to be involved in offensive and defensive roles [39], that may underpin the finding of the current study, that the highest number of ACC actions were performed by FB. However, none of the justifications can be confirmed as the total distance (TD) covered and HIR and ball possession were not included in the analysis. Therefore, such examination should be considered in future studies.

Similar to ACC actions, the frequency of DEC actions during matches was position-specific. More specifically, the number of DEC actions performed by CM, FB, and WM were higher than CB, which is consistent with previous studies [2, 26], although Oliva-Lozano, Fortes [24] found no differences in those actions in CB compared to other PP, except for WM. Those inconsistent findings observed between studies may be attributed to different playing formations as positional differences in high-intensity ACC and DEC actions were considerably affected by playing formations [17]. For instance, Tierney, Young [13] found that FB in a 3-5-2 formation executed a 20% higher number of DEC actions compared to those in a 4-4-2 formation. However, investigating the effect of formations on physical demands seems to be challenging as a tactical evolution in match-play suggests that teams do not tend to adopt only one specific formation throughout the whole match, given various situational factors affecting match performance [14]. Nonetheless, a holistic approach that amalgamates physical efforts with the tactical purpose of the action still seems to be required to provide a more comprehensive understanding of physical performance that includes ACC and DEC actions [40]. This may allow the quantification of such actions to be contextualised by ‘where’, ‘why’ and ‘how’ for each PP performing those actions during match-play [7]. This method may be practically useful when designing position-specific training drills.

### Match Location

Pertaining to ML, this study exhibited that ACC efforts of all players during home matches were higher than during away matches although small ES were observed. Further, DEC efforts between home and away matches did not differ significantly. Although ML has been reported to have less impact on the distance covered at various intensities than other contextual factors [3, 26], it may not be confirmed that this trend applies to ACC and DEC actions. One study that investigated the impact of ML on ACC and DEC efforts demonstrated that greater high-intensity ACC and DEC actions were observed in away matches rather than home matches [22], which is not consistent with the result of this study. Goalkeeper data was not omitted from analysis in the latter study, which may have decreased the mean ACC and DEC efforts reported, thus limiting a direct comparison with the current study. Furthermore, it may be argued that ML does not have a big effect on U23 teams compared to professional level. Likely, attendance of a crowd is lower at these games and there is more a focus on development than on the consequence of winning or losing a match [41]. This may translate in similarities in ACC and DEC demands in playing home or away. Given the paucity of information on the effect of ML on ACC and DEC efforts available in the previous literature [22], a definite conclusion cannot be drawn. Hence, future research that considers these limitations is warranted.

### Opposition Standard

The current study found the greatest number of ACC and DEC actions were observed in matches against higher standard opposition, but no significant difference was observed. This aligns with agrees with Rago, Silva [29] and Nobari, Ramachandran [28]. Generally, an increased density between players is expected when competing against better quality opponents, which may decrease the space available for players to run, which may predispose players to perform more high-intensity ACC and DEC actions as less time and space is available to make quick decisions to perform subsequent actions [27]. However, given the small number of player observations (low statistical power) in the current study and the research conducted by Rago, Silva [29], caution should be taken when generalising or interpreting GPS-derived ACC and DEC data.

### Interaction effect of PP, ML, and OS

The current study is the first to concurrently investigate an interaction effect of OS, ML, and PP on ACC and DEC efforts. One of the main findings was that players in a central position (i.e., CB, CM, and CF) performed more high-intensity actions in games against top and middle teams at home than away. At the same time, wide players (i.e., FB and WM) performed less high-intensity actions against top teams at home than playing away. This result demonstrates the interaction between PP, OS, and ML. In home games and playing against top teams, the central players seem more engaged in high-intensity actions, whereas the wide players perform less of those efforts. This may be due to the decreased space available in the central area of the pitch by an increase in the density of opponents when playing against top teams amplified by playing at home. This may restrict the opportunity for CM to perform high-speed running, which causes these players to execute greater DEC efforts in a narrow space compared to matches against bottom teams [6]. Against bottom teams, the WM are more engaged in high-intensity efforts, again amplified by playing at home. Both of these observations, however, such a spatial constraint may be affected by the score-line [18]. This result demonstrates the interaction between PP, OS, and ML. In home games and playing against top teams, the central players engaged in high-intensity actions, whereas the wide players performed less of those efforts. The novel finding of the present study may provide sports scientists with a more comprehensive insight into designing individualised training programmes while considering PP, ML, and OS concurrently.

### Limitations

Some limitations of the current study exist. Firstly, the GPS data examined in this study was from a single English Premier Development League team, thus, caution must be considered when drawing any generalisations from this study, as varying leagues have different characteristics and playing styles that may affect the physical demands during match-play [19]. Thus, these findings may potentially be different to the current study. Secondly, it has been reported that GPS metrics have a high measurement error and variation when capturing high-intensity actions (especially ACC and DEC) [42, 43], despite being previously adopted in research [44]. Therefore, using a valid and reliable accelerometer-derived device known to be more sensitive to capture ACC and DEC actions is warranted for future research, that will help improve measurement accuracy and provide more reliable data for analysis. Furthermore, the current study did not consider physical variables such as TD, HSR, and sprinting, as well as tactical variables such as formation and possession when analysing ACC and DEC efforts, similar to previous research [1, 44]. Consequently, the study produced a limited understanding of contextualised ACC and DEC actions, given these variables are reported to affect physical demands during match-play [40, 45]. Therefore, it is warranted that these variables should be considered for future research. Finally, the contextual factor of match outcome was not employed in this study analysis which should be considered for future research, since it has been proven that match outcome can affect the number of ACC and DEC [44].

## CONCLUSIONS

In conclusion, the number of high-intensity efforts is influenced by playing position and match location. Playing position have a great impact on ACC and DEC efforts with large effects. The findings of this study confirm several of the hypotheses, except hypothesis (3) regarding OS. Sports scientists may utilise the findings of the current study to design position-specific physical conditioning training and individualised recovery sessions whilst considering PP, ML, and OS independently or concurrently. However, this may be of greater interest when contextualising ACC and DEC actions with tactical variables as this may help practitioners design more effective training.

## Conflict of interest

The authors declare no conflict of interest.
